# Breast cancer mortality in synchronous bilateral breast cancer patients

**DOI:** 10.1038/s41416-019-0403-z

**Published:** 2019-02-26

**Authors:** Mathias Kvist Mejdahl, Jan Wohlfahrt, Marianne Holm, Eva Balslev, Ann Søegaard Knoop, Anne Tjønneland, Mads Melbye, Niels Kroman

**Affiliations:** 1 Department of Breast Surgery, Herlev and Gentofte Hospital, Copenhagen University Hospital, Herlev, Denmark; 2 Department of Epidemiology Research, Statens Serum Institut, Copenhagen, Denmark; 3 Danish Cancer Society Research Center, Copenhagen, Denmark; 4 Department of Pathology, Herlev Hospital, Copenhagen University Hospital, Herlev, Denmark; 5 Department of Oncology, Rigshospitalet, Copenhagen University Hospital, Copenhagen, Denmark; 6 Institute of Public Health, University of Copenhagen, Copenhagen, Denmark; 7 Department of Clinical Medicine, University of Copenhagen, Copenhagen, Denmark; 8 Department of Medicine, Stanford University School of Medicine, Stanford, CA USA; 9 Danish Cancer Society, Copenhagen, Denmark

**Keywords:** Cancer epidemiology, Epidemiology, Breast cancer

## Abstract

**Background:**

Evidence suggests that patients with synchronous bilateral breast cancer (SBBC), diagnosed within 4 months, have an inferior prognosis compared to unilateral breast cancer (UBC) patients. Using data from nationwide Danish clinical databases, this cohort study investigated whether the inferior prognosis could be explained by SBBC patients having a more aggressive disease, or whether the prognosis could be explained by the fact that they have two simultaneous cancers.

**Methods:**

Patients were diagnosed from 1999–2015. The main outcome was excess mortality, subtracting background population mortality from observed mortality. Differences between SBBC and UBC patients were evaluated by rate ratios (RR) and estimated by Poisson regression.

**Results:**

In total, 1214 SBBC and 59 177 UBC patients were included. SBBC patients had a significantly higher excess mortality than UBC patients after adjustment for age and period (RR = 1.73; 95% CI:1.44–2.08; *p* < 0.01) and after adjusting for characteristics of the worst tumour as traditionally done (RR = 1.31; 95% CI:1.08–1.57; *p* = 0.01). However, adjusting for characteristics of both tumours, using a more advanced competing risks model, no difference was observed (RR = 1.01; 95% CI:0.83–1.22; *p* = 0.93).

**Conclusions:**

Our study does not support that the inferior prognosis in SBBC patients is due to having more aggressive tumours per se, but rather the combined effect of having two simultaneous cancers.

## Background

Synchronous bilateral breast cancer (SBBC) is breast cancer diagnosed more or less simultaneously in both breasts in the same patient. The cut-off for synchronicity described in the literature has usually been between 3 and 6 months.^1^ Evidence supports viewing the two tumours in synchronous breast cancers as two primary lesions and not as one disease with metastatic spread.^2,3^ When comparing the prognosis of SBBC to that of unilateral breast cancer (UBC), in most previous work, the method has been to choose an index tumour, usually the prognostically worst, the largest, or the first to be diagnosed, based on which to compare the SBBC patient to the UBC patient.^4–15^ A meta-analysis found a higher breast cancer mortality among SBBC patients compared with UBC patients, with a pooled hazard ratio (HR) of 1.36 (95% CI 1.24–1.50).^1^ The approach of comparing SBBC to UBC using the worst tumour as index in the SBBC patient makes sense as the clinical consensus regarding the decision of adjuvant treatment for SBBC patients today is based on the most adverse tumour characteristics.^9^ But this approach does not offer an explanation as to why SBBC patients potentially have an inferior prognosis.

Our hypothesis was that the unexplained higher risk of breast cancer death seen among SBBC patients when comparing them to UBC patients based on their prognostically worst tumour could be explained by the combined risk of the two tumours. The aim of the current study was to analyse the prognosis of SBBC compared to UBC in a large and nationwide cohort of Danish women with detailed information on disease characteristics from both tumours.

## Methods

### Registries

The Danish Breast Cancer Group (DBCG) database is a nationwide clinical breast cancer database initiated in 1977.^16^ Previous quality assessments of the database have shown a near complete registration of breast cancer cases.^17,18^ Using the unique civil registration number assigned to all Danish citizens, info on diagnosis from the DBCG was linked to the Danish Pathology Register, which contains detailed information on all human tissues examined by a pathological department in Denmark.^19^ Information on death and emigration was obtained by linkage with the Civil Registration System.^20^

### Study population

All patients diagnosed with primary breast cancer in Denmark from 01 January 1999 to 31 December 2015 were identified in the DBCG database and screened for inclusion in the present study. January 1999 was chosen as cut-off, as the Danish Pathology Register is increasingly incomplete before this date. If patients were diagnosed with bilateral breast cancer, with the two cancers diagnosed within 4 months of each other, they were defined as SBBC patients. The cut-off was in accordance with another research group examining metachronous bilateral breast cancer in Denmark.^21^ The exclusion criteria were patients with disseminated cancer at time of diagnosis, locally advanced cancer, only ductal carcinoma in situ, previous malignancies (non-melanoma skin cancer excluded), patients receiving neoadjuvant therapy, patients not receiving surgical treatment, cancer of non-breast parenchyma origin, or age <18 years. Further, SBBC patients were excluded if they could not be identified with a breast cancer in the Pathology Register or had occult cancer on one side (e.g. axillary lymph node metastasis, but with no identified primary tumour in the breast). SBBC patients otherwise not having disseminated or locally advanced disease, were also excluded if the pathologist specifically had described one side as likely being a metastasis from the contralateral breast, as both UBC and SBBC patients with metastatic disease were excluded from the study.

### Disease characteristics and treatment

For UBC patients, data on patient characteristics, disease characteristics, and mortality was retrieved from the DBCG database. With SBBC patients, the policy has been only to register the patients in the database and subsequently let them go off-study. Thus, except for data on mortality and patient characteristics, no follow-up data is available on this patient group, and data on clinical and histopathologic characteristics are inconsistent in the DBCG database. For SBBC patients, detailed histopathologic information was therefore retrieved from the Danish Pathology Register.

Data on intention-to-treat (ITT) adjuvant protocol treatment was for UBC patients retrieved from the DBCG database. In Denmark, the DBCG publishes national multidisciplinary guidelines for diagnostic, follow-up and treatment of breast cancer,^22^ and patients are allocated to treatment protocols based on their risk profile. For UBC patients who were not included in a DBCG protocol and without missing data on disease characteristics, adjuvant ITT treatment was estimated based on DBCG treatment algorithms. Adjuvant treatment of SBBC patients is not registered in the DBCG database and was therefore defined according to DBCG treatment guidelines, and the general practice in Denmark for treatment of SBBC patients. In Denmark, the consensus is to perform risk stratification of SBBC patients based on the worst disease characteristics regardless of side. In the multivariable analyses, surgery and ITT radiotherapy were combined in a variable with three levels: mastectomy, mastectomy and radiotherapy, and breast conserving surgery (BCS) and radiotherapy. Supplement 1 shows a table with description, categorisation, and source of the data included in the study.

### Statistical analysis

Differences in histopathologic characteristics between SBBC and UBC patients were evaluated using logistic and multinomial regression analyses, with correlation between the right and left tumour in the same SBBC patient accounted for by a Generalised Estimating Equation correlation structure.

The excess mortality, where the observed mortality is subtracted from the expected mortality based on background population mortality rates, was used as a measure of breast cancer related death. The difference in excess mortality between women with SBBC compared to UBC was evaluated by rate ratios (RR) estimated by Poisson regression with expected mortality as offset using PROC NLMIXED in SAS. The statistical model is described in detail in supplement 2. The expected mortality was calculated by multiplying the person years at risk according to age (1 year) and time-period (5 years) with population mortality rates from Statistics Denmark. Patients were followed from primary surgery until death, emigration or end of follow-up (01 August 2017). For SBBC patients, first surgery date was used if surgery of the two tumours was not performed on the same day.

The excess mortality was adjusted in several ways. Initially, a model adjusting only for time-period (5-year intervals), age (5-year intervals), and time since diagnosis (1-year intervals) for both UBC and SBBC patients was applied. Subsequently, disease characteristics (tumour size, malignancy grade, histological subtype, nodal involvement, and ER-status) and treatment variables (ITT chemotherapy, and surgery/ITT radiotherapy) were added. Adjustment in UBC patients was performed using a traditional log-linear modelling of the rate by disease characteristics and treatment. For SBBC patients, adjustment was performed using three different ways to define the characteristics of the two tumours: the characteristics of the worst tumour, the worst disease characteristics regardless of side, and the characteristics of both tumours. Using the characteristics of the worst tumour, the rate for SBBC patients was modelled by a log-linear approach, and the disease characteristic and loco-regional therapy in a SBBC patient was defined by selecting the largest tumour, and if equal size, then by axillary lymph node metastases, histological subtype, and finally by tumour grade. Using the worst disease characteristics, the disease characteristic in a SBBC patient was defined by the disease characteristic of the à priori worst prognostic outcome regardless of side. If loco-regional treatment between the two sides differed in the SBBC patients, mastectomy and radiotherapy were chosen above the other loco-regional treatments, and mastectomy above BCS and radiotherapy. Finally, using the characteristics of both tumours, the two tumours in a SBBC patient were treated as competing risk for the excess mortality in the Poisson model, i.e., the rate in SBBC patients was modelled as a sum of two components both modelled by an exponential function of a linear form of the covariates for the two tumours.

Observed cumulative mortality rates were calculated as number of events divided by person years at risk in 1-year intervals and cumulated by 1 year intervals and predicted cumulative mortality rates were calculated using the model based predicted number of events.

To address missing data, a multiple imputation (MI) analysis by fully conditional specification methods was used to predict values of missing data (see supplement 3). A total of 20 imputations were performed. Using Rubin’s MI strategy, the imputed data sets were combined to produce inferences for the parameters in the models.

Data was analysed using SAS version 9.4 (SAS institute Inc., Cary, USA).

## Results

**Fig. 1:**
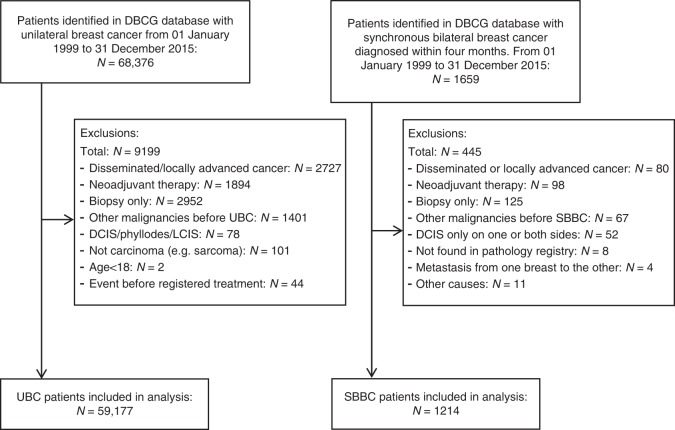
Flow chart. *DBCG* Danish Breast Cancer Group, *DCIS* ductal carcinoma in situ, *LCIS* lobular carcinoma in situ, *SBBC* synchronous bilateral breast cancer, *UBC* unilateral breast cancer

In the DBCG database, from 01 January 1999 to 31 December 2015, 1 659 patients were registered with SBBC. Among these, 1 214 SBBC patients were included in the study. For UBC patients, 59 177 patients out of 68 376 were included. Figure 1 shows the flow chart of causes for exclusion. For disease and patient characteristics see Table 1, and for treatment characteristics see Table 2. SBBC patient were older than UBC patients, and the distribution of all disease characteristics were statistically different from UBC patients, with the worst tumour in SBBC patients generally being larger and with more nodal involvement, and more often ER positive and lobular. SBBC patients more often received a mastectomy and were less often allocated to chemotherapy and radiotherapy.

**Table 1:** Patient and disease characteristics of unilateral and synchronous bilateral breast cancer patients

	Unilateral breast cancer		Synchronous bilateral breast cancer				*p*-value^a^
Total, *N*	59177		1214				
Age, *N* (%)							<0.01
<40 years	2219	(3.8)	16		(1.3)		
40–49 years	8051	(13.6)	92		(7.6)		
50–59 years	15164	(25.6)	214		(17.6)		
60–69 years	18275	(30.9)	412		(33.9)		
≥70 years	15468	(26.1)	480		(39.5)		
Menopausal status, *N* (%)							<0.01
Pre-menopausal	14362	(24.4)	164		(13.6)		
Post-menopausal	44619	(75.6)	1043		(86.4)		
Missing (% missing)	196	(0.3)	7		(0.6)		

	Unilateral		Worst tumour^b^		Contralateral tumour		
	*N*	%	*N*	%	*N*	%	
Histology							<0.01
Ductal	47931	81.2	956	78.7	959	79.0	
Lobular	6417	10.9	189	15.6	166	13.7	
Other	4709	8.0	69	5.7	89	7.3	
Missing (% missing)	120	(0.2)	0	(0.0)	0	(0.0)	
Oestrogen receptor status							<0.01
Positive	48801	83.3	1083	89.7	1107	92.9	
Negative	9792	16.7	125	10.3	85	7.1	
Missing (% missing)	584	(1.0)	6	(0.5)	22	(1.8)	
Combined HER2− & ER− status (≥01 Jan 2007)	33682	56.9	649	53.5	649	53.5	<0.01
ER+/HER2−	24315	76.7	501	81.1	553	90.4	
ER+/HER2+	2828	8.9	56	9.1	26	4.2	
ER− /HER2+	1425	4.5	22	3.6	9	1.5	
ER− /HER2−	3124	9.9	39	6.3	24	3.9	
Missing (% missing)	1990	(5.9)	31	(4.8)	37	(5.7)	
Malignancy grade							<0.01
I	16035	30.7	371	33.9	507	48.1	
II	23511	45.1	532	48.7	465	44.1	
III	12630	24.2	190	17.4	82	7.8	
Missing (% missing)	7001	(11.8)	121	(10.0)	160	(13.2)	
Tumour size							<0.01
0–20 mm	36596	62.2	553	45.8	1052	89.0	
21–50 mm	20440	34.7	593	49.1	126	10.7	
>50 mm	1798	3.1	62	5.1	4	0.3	
Missing (% missing)	343	(0.6)	6	(0.5)	32	(2.6)	
Nodal involvement							<0.01
0 metastases	32152	55.9	548	47.0	847	74.0	
1–3 metastases	17090	29.7	403	34.6	232	20.3	
4–9 metastases	5170	9.0	127	10.9	49	4.3	
>9 metastases	3131	5.4	88	7.5	17	1.5	
Missing (% missing)	1634	(2.8)	48	(4.0)	69	(5.7)	

*ER-status* oestrogen receptor status, *HER2-status* human epidermal growth factor receptor 2 status, *SBBC* synchronous bilateral breast cancer, *UBC* unilateral breast cancer, *y* years

^a^Test for difference in the distribution of disease and patient characteristics between unilateral and synchronous bilateral breast cancer patients using logistic and multinomial regression analyses. For bilateral breast cancer patients, information on disease characteristics from both sides were used, taking account of within patient correlation using a GEE correlation structure. Excluding missing values

^b^The worst tumour was selected based on size, then nodal involvement, then histological subtype, and then malignancy grade

**Table 2:** Treatment characteristics based on DBCG treatment guidelines for unilateral and treatment-consensus for bilateral breast cancer patients

	Unilateral breast cancer		Synchronous bilateral breast cancer			
			Worst tumour		Contralateral tumour	
	*N*	%	*N*	%	*N*	%
Surgery						
Mastectomy	25900	43.8	746	61.5	667	55.0
Breast conserving surgery	33277	56.2	468	38.6	545	45.0
Missing (% missing)	0	(0.0)	0	(0.0)	2	(0.2)
Radiotherapy^a^						
Yes	40965	76.4	639	63.1	580	57.3
No	12685	23.6	374	36.9	433	42.7
Missing (% missing)^b^	5527	(9.3)	201	(16.6)	201	(16.6)
Chemotherapy^a^			N		%	
Yes	22236	41.4	335		33.1	
No	31430	58.6	678		66.9	
Missing (% missing)^b^	5511	(9.3)	201		(16.6)	
Endocrine therapy^a^						
Yes	36023	67.1	883		87.2	
No	17643	32.9	130		12.8	
Missing (% missing)^b^	5511	(9.3)	201		(16.6)	

*DBCG* Danish Breast Cancer Group, *SBBC* synchronous bilateral breast cancer, *UBC* unilateral breast cancer

^a^For UBC patients, intention-to-treat allocation based on DBCG protocols is shown. For SBBC patients, adjuvant treatment is estimated based on consensus-treatment for complete cases

^b^For SBBC patients, missing data is due to non-complete cases for tumour characteristics, and for UBC patients missing data is for patients not included in a DBCG protocol

**Fig. 2:**
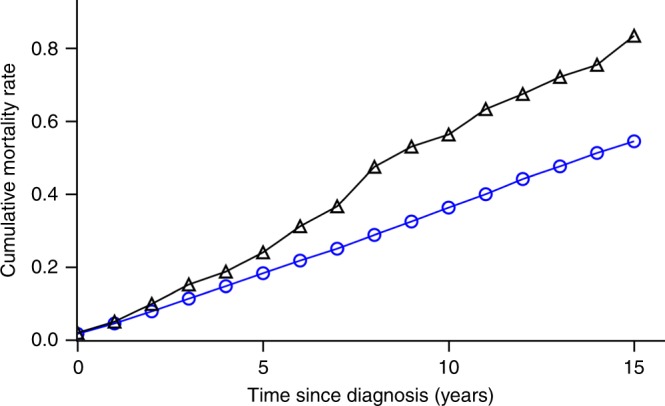
Observed cumulative mortality rates of synchronous bilateral () and unilateral () breast cancer patients

SBBC patients contributed 8 924-person years at risk with 454 patients dying during follow-up. UBC patients contributed 465 090-person years at risk, and 16 096 patients died during follow-up. The median follow-up time for the entire cohort was 7.23 years (IQR: 3.97–11.10), with slightly shorter follow-up time for SBBC patients (median: 6.79 years) than UBC patients (median: 7.24 years). The 5- and 10-year overall survivals for SBBC patients were 80.7% (95% CI: 78.3–82.9%) and 56.7% (95% CI: 53.3–60.0%), and for UBC patients 85.1% (95% CI: 84.8–85.4%) and 70.5% (95% CI: 70.0–70.9%). In supplement 4, the Kaplan-Meier plot with 95% CI bands is shown for SBBC and UBC patients, and Fig. 2 shows the observed cumulative mortality rates for SBBC and UBC patients.

**Fig. 3:**
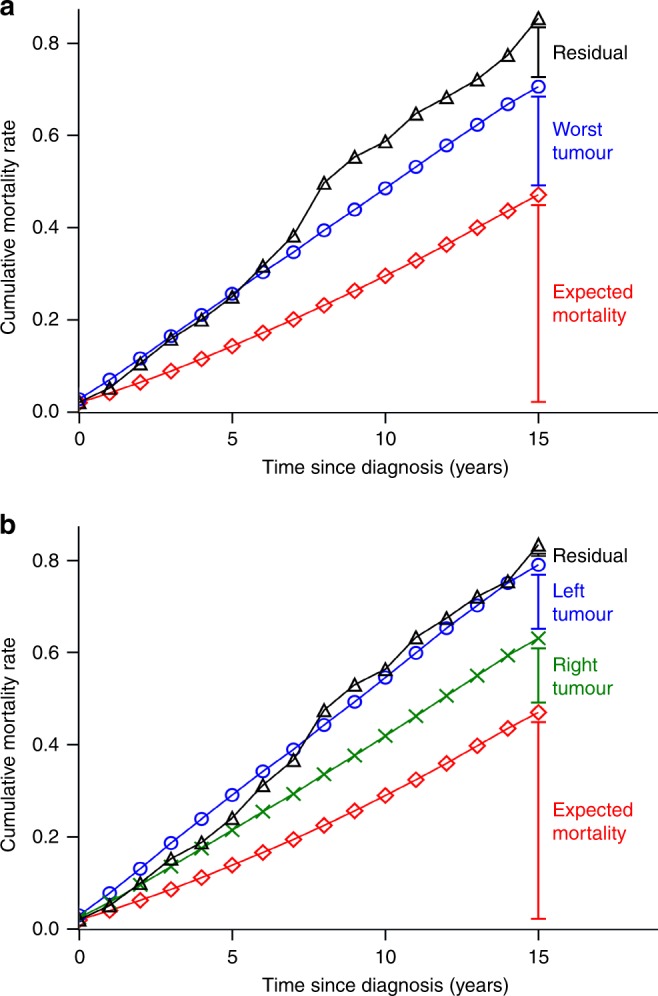
Observed and predicted cumulative mortality rates (CMR) of synchronous bilateral breast cancer patients. **a** Predicted rates based on characteristics of the worst tumour: Observed CMR (), Predicted CMR due to characteristics of the worst tumour (+ expected) (), and expected background CMR (). **b** Predicted rates based on the characteristics of both tumours: Observed CMR (), Predicted CMR due to left tumour (+ expected + right) (), Predicted CMR due to right tumour (+ expected) (), and expected background CMR ()

The RR for excess mortality of SBBC compared to UBC was 1.73 (95% CI: 1.44–2.08; *p* < 0.01) when adjusting for age, time-period, and time since diagnosis (see Table 3), i.e. the mortality, beyond the level in the population, was 73% higher in SBBC patients compared to UBC patients. Adjusting for the characteristics of the worst tumour, the RR was 1.31 (95% CI: 1.08–1.57; *p* = 0.01) and adjusting for the worst disease characteristics regardless of side, the RR was 1.17 (95% CI: 0.97–1.42; *p* = 0.10). Figure 3a shows the predicted cumulative mortality rates for SBBC patients estimated based on the characteristics of the worst tumour plotted against the observed cumulative mortality rates. In patients with a risk profile that would elicit chemotherapy, the RR was 1.37 (95% CI: 1.03–1.83), and for patients that should not receive chemotherapy, the RR was 1.05 (95% CI: 0.81–1.37). The two RRs were not significantly different (*p* = 0.18).

**Table 3:** Rate ratios of excess mortality comparing synchronous bilateral breast cancer patients to unilateral breast cancer patients

Poisson regression adjusting for age, time since diagnosis, and time-period^a^		Rate Ratio (95% CI)	*P*-value
Disease adjustment	Treatment adjustment		
No	No	1.73 (1.44–2.08)	<0.01
Characteristics of worst tumour^b^	Yes^b^	1.31 (1.08–1.57)	0.01
Worst characteristics regardless of side^c^	Yes^c^	1.17 (0.97–1.42)	0.10
Both tumours^d^	Yes^d^	1.01 (0.83–1.22)	0.93

^a^Adjustment for age in 5-year intervals, for time since diagnosis in 1-year intervals, and period in 5-year intervals

^b^Adjusting for the worst tumour and treatment. The worst tumour was selected based on size, then nodal involvement, then histological subtype, and then tumor grade. If nodal involvement was used before size for selection of the worst tumour, the RR was 1.28 (95% CI: 1.06–1.53)

^c^Adjusting for the worst tumour characteristics and treatment. The worst tumour characteristics were selected regardless of side (e.g. if left tumour was 20 mm and ER negative, and right tumour was 55 mm and ER positive, 55 mm would be selected for size and ER negative for ER-status)

^d^Adjusting for right tumour, left tumour, and treatment

In Fig. 3b the predicted cumulative rates for expected mortality and the contribution of the right and left tumour on excess mortality were plotted against the observed cumulative mortality rates. The residual effect of bilateral disease when adjusting for characteristics of both tumours was RR = 1.01 (95% CI: 0.83–1.22; *p* = 0.93).

The results were similar when using imputation to take missing characteristics into account (see supplement 3).

## Discussion

In the present study we found that the excess mortality is around 30% higher among SBBC patients compared to UBC patients when the comparison is based on characteristics of the prognostically worst tumour. The worst tumour in the SBBC patient is more often larger, and with more nodal involvement than the disease in the UBC patient explaining why adjustment for these characteristics changes the point estimate from 1.73 to 1.31. In the model comparing SBBC patients to UBC patients based on the most adverse disease characteristics regardless of side, the excess mortality was around 20% higher among SBBC patients, although not reaching statistical significance. In the ‘two tumour model’, the excess mortality among SBBC patients could be attributed to the combined mortality of the two cancers.

The excess mortality in SBBC patients when adjusting for the characteristics of the worst tumour is comparable with previous findings. The meta-analysis on the impact of SBBC on breast cancer mortality showed a more than 30% higher mortality of SBBC compared to UBC.^1^ This result was especially driven by one larger study using data from the Surveillance, Epidemiology, and End Results database from the NIH.^1,23^ This study showed a relative risk for breast cancer death around 1.45 when comparing SBBC to UBC.^23^ Patients were diagnosed from 1973–2000, and for disease characteristics, only adjustment for histological subtype and stratification on stage was performed.^23^ Two other large studies, where patients were also diagnosed before 2000, reported similar results,^10,11^ and one large (*n* = 837) and more recent study, that included adjustments for more detailed disease characteristics, reported a hazard ratio of 1.17 (95% CI: 0.91 to 1.51), which is slightly lower than the findings from the present study.^4^ During the last 30 years the prognosis of breast cancer has improved substantially, and the treatment of breast cancer has also changed considerably with more adjuvant treatment and less extensive surgery.^16^ The generalisability of the results from studies with patients diagnosed in the 90’s and earlier to patients today is therefore questionable. Further, these earlier studies only made rather crude adjustments for disease characteristics.^10,11,23^ As shown in the present study, the effect of SBBC on excess mortality changes considerably when adjustments for detailed disease characteristics are included.

When adjusting for disease characteristics of both tumours we found no excess mortality in SBBC patients. The hypothesis of viewing SBBC as a simple competing risk model with two breast cancers ‘competing to be fatal to the patient’ is very plausible. The model does however assume that the effects on breast cancer mortality attributed by each of the two tumours in a SBBC patient are independent of each other. So hypothetically, if we radically removed only the left tumour by surgery and did not treat the right tumour, this procedure should not influence the natural course of the right tumour. Although it is a strong assumption, viewing the tumours as acting independently on breast cancer mortality appears to be a good approximation of the prognosis of bilateral breast cancer patients in the present study. Two studies have tried to analyse the prognosis incorporating information from both tumours, one by describing the effect of concordance in tumour stage on prognosis,^23^ and another describing the effect of concordance in oestrogen receptor status on prognosis.^24^ These studies showed that characteristics of both tumours are of importance in determining the prognosis for a SBBC patient, and the present study further adds to this assumption by showing that the two tumours each has the same effect on the excess mortality as an equivalent unilateral cancer. The reason why the ‘worst tumour model’ only gives a 30% higher excess mortality and not a double risk, is because SBBC patients usually present with a large index tumour and a smaller contralateral tumour as supported by the present data. In Denmark it is a standard procedure to perform bilateral mammography and ultrasound when a breast cancer is diagnosed, and a subclinical contralateral lesion would therefore usually be detected during diagnostic evaluation. In a study on the incidence of bilateral breast cancer, Hartman et al. showed that the incidence of SBBC was higher than what could be explained by chance, and they hypothesised that a more susceptible group of women were involved in SBBC.^25^ Also, a study by Kwast et al. showed that women with bilateral breast cancer had a higher risk of a third primary cancer of non-breast origin.^26^ Based on the current findings, one could argue that the increased risk of a third primary disease and the potential susceptibility to breast cancer formation does not seem to have an impact on the excess mortality in itself, as the excess mortality could be explained by the two breast cancer lesions in the SBBC patient.

It has been suggested that SBBC patients might not be optimally treated with adjuvant therapy,^1^ and that bilateral disease should be added as a prognostic indicator when determining allocation to adjuvant treatment.^1,13^ The ‘worst characteristics model’ only gives a slightly increased excess mortality (RR = 1.17), although not reaching statistical significance, and when dividing SBBC patients based on allocation to chemotherapy, no difference in the impact of bilateral disease on excess mortality could be observed. It should be noted though, that these findings are limited by a lack of power.

The present study is one of the largest studies to date on the prognosis of SBBC, and the largest with detailed information on disease characteristics. The use of excess mortality could both be viewed as a strength and a limitation. Establishing the cause of death can be difficult, and autopsies are not performed on a routine basis in Denmark. On the other hand, when using excess mortality, it is assumed that the cause of death we are investigating is rare in the general population as these deaths are included in the deaths observed in the general population. One limitation is that we did not have definitive information on adjuvant treatment. Instead treatment was estimated based on DBCG guidelines and for SBBC patients on treatment-consensus. But adherence to guidelines of breast cancer treatment in Denmark is considered high.^22^ Another limitation is missing data potentially introducing bias. This was addressed in our multiple imputation analysis. Here the RR for excess mortality of SBBC was attenuated slightly, although the interpretation of the results did not change.

In conclusion, the present study has shown that patients with SBBC have a 30% higher excess mortality than UBC patients when the comparison is based on the largest tumour in the SBBC patient, but when taking the characteristics of both tumours into account, no difference in excess mortality between SBBC and UBC patients was found. The study does therefore not support that SBBC tumours per se are more aggressive than tumours in UBC patients.

## Supplementary information

Supplement 2: Estimation of excess mortality rate ratios

Supplement 3: Multiple Imputation Analysis

Supplement 1: Information obtained from registries

Supplement 4: Kaplan-Meier Curve for overall survival

## Data Availability

The data that support the findings of this study are archived at governmental institutions in Denmark and can be obtained through application to the relevant data agencies. Data from the Danish Breast Cancer Group Database is available through Danish Clinical Registries (RKKP), and data from the Danish Pathology Register is available through the Health Data Agency. Data on population mortality rates in age groups and calendar periods, and according to gender, are publicly available at Statistics Denmark (http://www.statistikbanken.dk/statbank5a/default.asp?w=1920).
