# Supplementary material for: Breast cancer mortality in synchronous bilateral breast cancer patients
Source: Br J Cancer. 2019 Feb 26;120(7):761–7. doi: 10.1038/s41416-019-0403-z (PMC6461871; doi:10.1038/s41416-019-0403-z)
Supplement: Supplementary file 1 — Supplement 2: Estimation of excess mortality rate ratios [file 41416_2019_403_MOESM1_ESM.docx]

**Supplement 2: Estimation of excess mortality rate ratios**

Initially, the number of deaths (*x_i_*) and observation time (*T_i_*) were summed within strata of age, period, time since diagnosis, disease characteristics, and treatment, with *i* being an index for these strata.

*x_i_* was analysed by Poisson regression with mean parameter *μ_i_. μ_i_* was modelled as

*μ_i_* = *T_i_ λ_i_* = *T_i_ (λ_expected,i_ + λ_excess,i_)* = *E_i_ + T_i_ λ_excess,i_*

with *T_i_* and *λ_expected,i_* considered known, *λ_expected,i_* taken as the population mortality rates from Statistics Denmark based on age (1 year categories) and period (5 years categories), and *E_i_* as the expected mortality in strata *i* treated as an offset in the analysis.

*λ_excess,i_* was modelled in four different ways. The four models are described below with the following notation: *x_bilat(i)_* is an indicator variable equal to 1 in synchronous bilateral breast cancer (SBBC) patients. *a(i)*, *β_a_* and *x_a(i)_*, respectively, are index, parameters and indicator variables for age, period, and time since diagnosis categories. *b(i)* and *β_b_*, respectively, are index and parameters for disease characteristics (tumour size, malignancy grade, histological subtype, nodal involvement, and ER-status) and treatment variables (ITT chemotherapy, and surgery/ITT radiotherapy). The index *a(i)* and *b(i)* do not include the reference categories.

*Model 1*: no adjustment for disease characteristics or treatment:

*λ_excess,i_* = *exp*(*β_1,bilat_ x_bilat(i)_* + ∑*_a_* (*β_a_x_a(i)_*))

RR = *exp(β_1,bilat_)* from this model is reported in table 3 with label “Disease adjustment: no. Treatment adjustment: no”.

*Model 2*: adjustment for characteristics of worst tumour and treatment:

*λ_excess,i_* = *exp* (*β_2,bilat_ x_bilat(i)_* + ∑*_a_* (*β_a_x_a(i)_*) + ∑*_b_* (*β_b_x_wt,b(i)_*))

*x_wt,b(i)_* are indicator variables for characteristics of the worst tumour and treatment categories for category *b(i)*. For unilateral breast cancer (UBC) patients, *x_wt,b(i)_* are characteristics of the single (unilateral) tumour.

RR = *exp(β_2,bilat_)* is reported in table 3 with label “Disease adjustment: Characteristics of worst tumour. Treatment adjustment: yes”.

*Model 3*: adjustment for worst disease characteristics regardless of side and treatment:

*λ_excess,i_* = *exp* (*β_3,bilat_ x_bilat(i)_* + ∑*_a_* (*β_a_x_a(i)_*) + ∑*_b_* (*β_b_x_wc,b(i)_*))

*x_wc,b(i)_* are indicator variables for worst disease characteristics and treatment categories for category *b(i)*. For UBC patients, *x_wc,b(i)_* are characteristics of the single tumour.

RR = *exp(β_3,bilat_)* is reported in table 3 with label “Disease adjustment: Worst characteristics regardless of side. Treatment adjustment: yes”.

*Model 4*: adjustment for characteristics of both tumours and treatment:

*λ_excess,i_* = *exp* (*β_4,bilat_ x_bilat(i)_* + ∑*_a_* (*β_a_x_a(i)_*) + ∑*_b_* (*β_b_x_lt,b(i)_*)) + *exp* (*β_4,bilat_ x_bilat(i)_* + ∑*_a_* (*β_a_x_a(i)_*) + ∑*_b_* (*β_b_x_rt,b(i)_*))

= *exp* (*β_4,bilat_ x_bilat(i)_* + ∑*_a_* (*β_a_x_a(i)_*)) (*exp* (∑*_b_* (*β_b_x_lt,b(i)_*)) + *exp* (∑*_b_* (*β_b_x_rt,b(i)_*)))

*x_lt,b(i)_* are indicator variables for characteristics and treatment of the left tumour. *x_rt,b(i)_* are indicator variables for characteristics and treatment of the right tumour. For chemotherapy, the indicator variables are similar for left and right tumour. The model is based on the assumption that the tumours in the two breasts are two independent competing causes of excess mortality. For UBC patients, *x_lt,b(i)_* and *x_rt,b(i)_* are zero for the breast without a tumour.

RR = *exp(β_4,bilat_)* is reported in table 3 with label “Disease adjustment: Both tumours. Treatment adjustment: yes”.

Using model 4 for *λ_excess,i_* the model for *μ_i_* is

*μ_i_* = *E_i_ + T_i_ (exp* (*β_4,bilat_ x_bilat(i)_* + ∑*_a_* (*β_a_x_a(i)_*)) (*exp* (∑*_b_* (*β_b_x_lt,b(i)_*)) + *exp* (∑*_b_* (*β_b_x_rt,b(i)_*))))

All models were based on the assumption of similar effects of disease characteristics and treatment for SBBC and UBC patients, i.e. the parameters *β_b_* were used in both patient groups. To evaluate this assumption, models allowing for different effects for UBC and SBBC patients were fitted (data not shown), and no significant differences in effects were observed using likelihood ratio tests.

For all models the parameters were estimated using procedure NLMIXED in SAS.
