# Supplementary material for: Breast cancer mortality in synchronous bilateral breast cancer patients
Source: Br J Cancer. 2019 Feb 26;120(7):761–7. doi: 10.1038/s41416-019-0403-z (PMC6461871; doi:10.1038/s41416-019-0403-z)
Supplement: Supplementary file 2 — Supplement 3: Multiple Imputation Analysis [file 41416_2019_403_MOESM2_ESM.docx]

**Supplement 3: Multiple imputation analysis**

Estimation of ITT treatment was done before a multiple imputation (MI) analysis was performed. For unilateral breast cancer (UBC) patients not in protocol who originally had missing data, it was assumed that adjuvant treatment would not have been allocated to the patient. For synchronous bilateral breast cancer (SBBC) patients this was assumed if both sides had missing data on a given disease characteristic. If data was only missing on one side, the non-missing value was chosen to estimate ITT treatment.

To address missing data, a MI analysis by fully conditional specification methods was used to predict values of missing data. Imputations were performed for disease characteristics. Other tumour characteristics, age, person years at risk, and event were used to predict missing values. For SBBC patients, imputation was also based on disease characteristics from the contralateral disease. A total of 20 imputations were performed. Using Rubin’s MI strategy, the 20 imputed data sets were combined to produce inferences for the parameters in the models. The multiple imputation analyses were performed using PROC MI and PROC MIANALYZE in SAS.

A total of 201 (16.6%) patients with SBBC had missing data in at least one of the disease characteristics, and for UBC patients, 4310 (7.3%) had missing data. Among SBBC patients with missing data, 66 (32.8%) had missing on both sides, and for these patients it was assumed that no adjuvant treatment had been allocated. For the remaining 135 SBBC patients with missing data, 27 would be allocated to chemotherapy according to protocol, 60 would be allocated to radiotherapy of the left breast, and 63 of the right breast. In the model using the characteristics of the worst tumour, identification of the index lesion was first based on tumour size, then by nodal involvement, histologic subtype, and malignancy grade. If only one side had missing tumour size, the tumour with the indicated size was chosen as index. For the remaining 6 patients, the index tumour was chosen based on the imputed data. For UBC patients with missing data, 1938 (45.0%) were not in protocol, and they were assumed not to have received adjuvant treatment. Among 3573 UBC patients not in protocol but with otherwise complete data, ITT treatment was estimated based on DBCG protocol guidelines, and 613 (17.2%) were assumed to be allocated to chemotherapy, and 1260 (35.3%) to radiotherapy.

In the MI analysis using the characteristics of the worst tumour, the RR for SBBC vs UBC was 1.26 (95% CI: 1.05-1.51; *p*=0.01). Estimates for the different MI data sets ranged between 1.24 and 1.29. In the model using the worst characteristics regardless of side, the RR for SBBC vs UBC was 1.10 (95% CI: 0.92-1.32; *p*=0.28). Estimates for the different MI data sets ranged between 1.08 and 1.13. Using the characteristics of both tumours, the RR was 0.94 (95% CI: 0.79-1.12; *p*=0.5182) ranging from 0.93 to 0.96 in the different MI data sets. The between variance was <0.0001 and within variance was 0.008 for all three models.
