# Supplementary material for: Breast cancer mortality in synchronous bilateral breast cancer patients
Source: Br J Cancer. 2019 Feb 26;120(7):761–7. doi: 10.1038/s41416-019-0403-z (PMC6461871; doi:10.1038/s41416-019-0403-z)
Supplement: Supplementary file 3 — Supplement 1: Information obtained from registries [file 41416_2019_403_MOESM3_ESM.docx]

**Supplement 1: Information obtained from registries**

| Supplementary table. Categorisation of variables and data source for information | | | |
| --- | --- | --- | --- |
|  | Description and/or categorisation of variables | Data source for Unilateral breast cancer patients | Data source for synchronous bilateral breast cancer patients |
| Identification of patients | - | DBCG | DBCG |
| Patient characteristics | | | |
| Menopausal status | Postmenopausal  Premenopausal | DBCG | DBCG |
| Follow-up and vital status | Date of death, end of follow-up, or emigration.  Vital status (dead or alive) | DBCG linkage to civil registration system | DBCG linkage to civil registration system |
| Exclusion criteria | | | |
| Dissemination/locally advanced | Disseminated or locally advanced  Not disseminated and not locally advanced | DBCG | DBCG and DPR |
| Other malignancies before breast cancer | Other malignancies before breast cancer (not including non-melanoma skin cancer) | DBCG | DPR |
| Neoadjuvant therapy | - | DBCG | DBCG and DPR |
| Disease characteristics | | | |
| Histology | Ductal  Lobular  Other  (exclusions: sarcomas, DCIS, LCIS or phyllodes tumours) | DBCG | DPR |
| Oestrogen Receptor status | Positive (≥10% from 1999 to April 2010 and ≥1% from April 2010 and onwards)  Negative (<10% before April 2010 and 0% from April 2010 and onwards) | DBCG | DPR |
| HER2 status | Positive if in situ hybridization was 3+ or 2+ if HER2/CEN17 ratio≥2.  Negative if 1+ or 2+ and HER2/CEN17 ratio<2. | DBCG | DPR |
| Malignancy grade | Grade I  Grade II  Grade III  (in multivariable analyses, other carcinomas were treated as grade I) | DBCG | DPR |
| Tumour size | ≤20mm  21-50mm  ≥51mm | DBCG | DPR |
| Nodal involvement | 0 metastases  1-3 metastases  4-9 metastases  ≥10 metastases | DBCG | DPR |
| Treatment | | | |
| Surgical treatment | Mastectomy  BCS | DBCG | DPR |
| Chemotherapy | Yes  No | DBCG: ITT chemotherapy if allocated according to DBCG protocol. If patients had missing data on protocol allocation, and otherwise no missing data, ITT chemotherapy was estimated based on DBCG treatment algorithms. | No direct ITT chemotherapy information available. Therefore, estimation of ITT chemotherapy was based on DBCG treatment algorithms, and SBBC patients were risk stratified based on the worst disease characteristic regardless of side. |
| Radiotherapy | Yes  No | DBCG: ITT radiotherapy if allocated according to DBCG protocol. If patients had missing data on protocol allocation, and otherwise no missing data, ITT radiotherapy was estimated based on DBCG treatment algorithms. | No direct ITT radiotherapy information available. Therefore, estimation of ITT radiotherapy was based on DBCG treatment algorithms, giving 4 possible treatment combinations: no treatment, treatment of right, treatment of left, and treatment of both. |
| Anti-HER2 treatment | Yes  No | DBCG: ITT anti-HER2 if allocated according to DBCG protocol. If patients had missing data on protocol allocation, and otherwise no missing data, ITT anti-HER2 was estimated based on DBCG treatment algorithms. | No direct ITT anti-HER2 treatment information available. Therefore, estimation of ITT anti-HER2 was based on DBCG treatment algorithms. |
| Endocrine treatment | Yes  No | DBCG: ITT endocrine therapy if allocated according to DBCG protocol. If patients had missing data on protocol allocation, and otherwise no missing data, ITT endocrine therapy was estimated based on DBCG treatment algorithms. | No direct ITT endocrine treatment information available. Therefore, estimation of ITT endocrine therapy was based on DBCG treatment algorithms. |
| Abbreviations: CEN17: centromere of chromosome 17; DBCG: Danish Breast Cancer Group; DCIS: Ductal Carcinoma In-situ; DPR: Danish Pathology Registry; HER2: Human Epidermal Growth factor Receptor 2; ITT: intention to treat; LCIS: Lobular Carcinoma In-situ. | | | |
