# Supplementary figures and images for: Breast cancer mortality in synchronous bilateral breast cancer patients
Source: Br J Cancer. 2019 Feb 26;120(7):761–7. doi: 10.1038/s41416-019-0403-z (PMC6461871; doi:10.1038/s41416-019-0403-z)

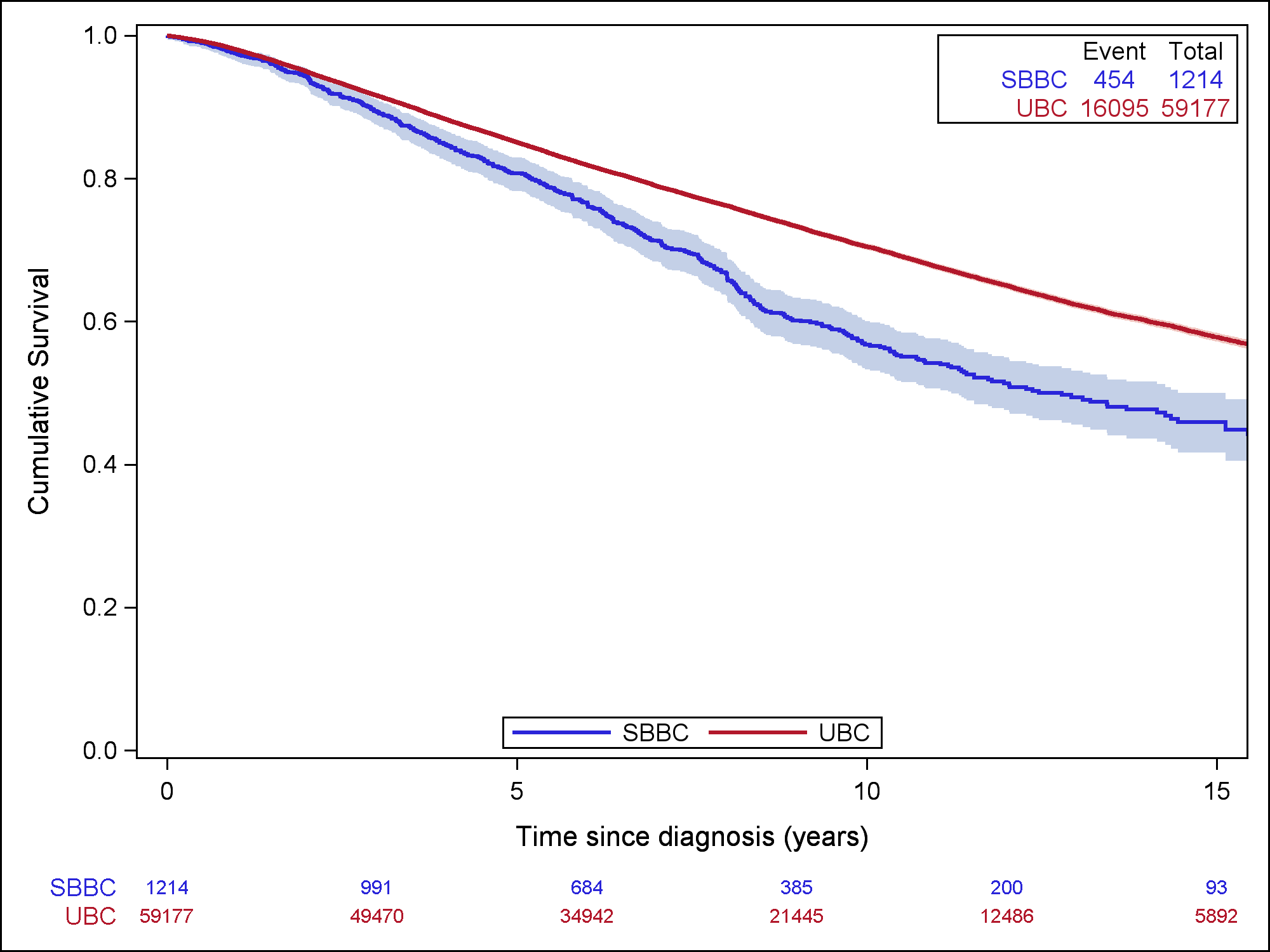

Supplement: Supplementary file 4 — Supplement 4: Kaplan-Meier Curve for overall survival [file 41416_2019_403_MOESM4_ESM.tif]
